# Targeting biofilm-related genes in a clinical methicillin-resistant *Staphylococcus aureus* isolate using CRISPR-Cas9 gene editing

**DOI:** 10.3389/fmicb.2026.1843899

**Published:** 2026-06-10

**Authors:** Aysegul Ates, Sohret Aydemir, Safak Ermertcan

**Affiliations:** 1 Department of Pharmaceutical Microbiology, Faculty of Pharmacy, University of Ege, Izmir, Türkiye; 2 Department of Medical Microbiology, Faculty of Medicine, University of Ege, Izmir, Türkiye

**Keywords:** antimicrobial resistance, biofilms, CRISPR-Cas9, gene editing, methicillin-resistant *Staphylococcus aureus*

## Abstract

**Background:**

Methicillin-Resistant *Staphylococcus aureus* (MRSA) is a major clinical challenge due to its biofilm-forming ability. Innovative therapeutic strategies are essential to prevent bacterial attachment and disrupt biofilm structures. This study investigates the potential of CRISPR technology as a tool to combat resistance by targeting the biofilm-associated genes *icaA*, *icaD*, and *bap* in MRSA.

**Methods:**

Specific guide RNAs were cloned into pCasSA plasmids to target the *icaA*, *icaD*, and *bap* genes. Gene expression changes were quantified using quantitative PCR (qPCR), and mutations were confirmed through Sanger sequencing. Biofilm formation was assessed by crystal violet assay, and antimicrobial susceptibility was evaluated by broth microdilution and disk diffusion methods.

**Results:**

qPCR analyses confirmed significant reductions in gene expression: 3.3-fold for *icaA*, 2.3-fold for *icaD*, and 1.7-fold for *bap*. Sanger sequencing confirmed point mutations and indels within the target regions of genes. Biofilm formation decreased markedly, with 6-fold in icaA-mutant, 5.6-fold in icaD-mutant, and threefold in bap-mutant strains. MIC values were substantially reduced in all mutant strains: oxacillin MIC decreased 64-fold, 16-fold, and 4-fold in *icaA*-, *icaD*-, and *bap*-mutants, respectively, and ciprofloxacin MIC decreased 64–128-fold. Zone diameters for cefoxitin, norfloxacin, and gentamicin increased up to twofold across all mutant strains. All strains remained resistant according to EUCAST clinical breakpoints.

**Conclusion:**

This study demonstrates that CRISPR-Cas9-mediated disruption of biofilm-associated genes is an effective strategy for inhibiting biofilm formation in MRSA. Targeted disruption of *icaA*, *icaD*, and *bap* significantly reduced biofilm formation and partially attenuated antimicrobial resistance phenotypes. These findings highlight the potential of pathogen-specific CRISPR-based anti-virulence strategies as complementary approaches for the treatment of biofilm-associated infections.

## 1 Introduction

Methicillin-resistant *Staphylococcus aureus* (MRSA) is a serious threat in both community and healthcare settings (Centers for Diseaese Control and Prevention (CDC), 2019; Shoaib et al., 2023). Its multidrug resistance and diverse virulence factors substantially limit therapeutic options and consequently complicate effective patient management (Abebe and Birhanu, 2023; Brdová et al., 2024). Biofilms of MRSA are microbial communities originating from cells firmly attached to biotic or abiotic surfaces (Patel and Rawat, 2023). Biofilms hinder antibiotic penetration and harbor dormant bacterial populations with markedly reduced antimicrobial sensitivity (Kaushik et al., 2024; Liu et al., 2024). Biofilm-associated infections are increasingly difficult to treat, leading to recurrent infections, prolonged hospital stays, and elevated mortality rates (Sahoo and Meshram, 2024; Wasfi et al., 2025). Consequently, the eradication of MRSA and its associated biofilms remains a critical priority. Targeting of biofilm associated genes in MRSA virulence is essential for developing novel therapeutic approach (Le and Otto, 2024; Badge et al., 2025; Subramani and Venugopal, 2025).

Given the urgent need for innovative antimicrobial strategies, CRISPR-Ca9 based technologies offer a promising avenue for developing targeted approaches against resistant pathogens (Ates et al., 2024). The targeted disruption of a gene significantly reduces its function, thereby enabling the exploration of CRISPR’s anti-virulence potential against bacterial infections. CRISPR-based anti-virulence strategies preserve the host microbiota by selectively targeting virulence or resistance determinants specific to pathogenic bacteria, unlike broad-spectrum antibiotics (Olatunji et al., 2024).

In this study, we utilized a complementary anti-virulence strategy by using CRISPR-Cas9 to disrupt biofilm structural genes (icaA, icaD, and bap). The icaA, icaD and bap genes play crucial roles in MRSA pathogenesis. The *ica*A and *ica*D genes are essential for intercellular adhesion and mature biofilm formation, while surface-associated proteins such as Bap also contribute to biofilm development (Armoon et al., 2024; Peng et al., 2022). The presence of *ica* genes is associated with enhanced virulence (Nguyen et al., 2020; Silva et al., 2021; Assefa and Amare, 2022; Peng et al., 2022). This study focuses to reduce biofilm-mediated resistance in clinical MRSA to improve treatment efficacy. To our knowledge, this was the first study to apply CRISPR-Cas9-mediated genome editing to *ica*A, *ica*D, and *bap* in a clinical MRSA isolate. We targeted *icaA*, *icaD*, and *bap* genes to suppress biofilm formation and evaluated the effects on antibiotic susceptibility. This study highlights the potential of CRISPR-Cas9 gene editing as a tool to functionally characterize bacterial virulence factors. This study suggest that combining CRISPR-mediated anti-virulence approaches with conventional antibiotics may offer a synergistic strategy to enhance treatment efficacy and mitigate resistance development, providing a rationale for future studies in this field.

## 2 Materials and methods

### 2.1 Bacteria, plasmid, oligos, primers

The study was conducted in accordance with the Declaration of Helsinki and approved by Medical Research Ethics Committee of Ege University (approval number: 24-3 T/39; date of approval: March 14, 2024). The study involved a clinical MRSA strain isolated from a blood sample of a patient hospitalized in the Cardiovascular Surgery Service. The isolation was performed on Columbia Agar with 5% Sheep Blood (COS agar) (Biomerieux, Lyon, France) by the Department of Medical Microbiology at Ege University Hospital. Identification and antibiotic susceptibility testing of the clinical MRSA isolate were carried out by Matrix-assisted laser desorption ionization-time of flight mass spectrometry (MALDI-TOF MS) (VITEK MS Prime, Biomerieux, Lyon, France) and VITEK-2 Compact (Biomerieux, Lyon, France) automated system, respectively. VITEK-2 test results were confirmed by broth microdilution testing. *S. aureus* ATCC 29213 was used as the control strain. Bacterial isolates were stored in Brain-Heart Infusion (BHI) broth (Merck KGaA, Darmstadt, Hessen, Germany) with 10% glycerin at −80 °C until use. pCasSA was provided by Quanjiang Ji (Addgene *#9*8211). The primers and oligonucleotides, detailed in Table 1, were specifically designed for the present work.

**Table 1:** Primers and oligos used in this study.

Oligonucleotide	Intended use	Primer	Sequence (5′-3′)	Amplicon lenghts
PRIMERS	Colony PCR	*ica*A-F	GAAAGTCGATTTACAAGAAAACAG	120 bp
		PCR-R	GGGTATGGACAGATCTCAAAAAAAGCAC	
		*ica*D-F	GAAATTAATAATCCAGTATACTGT	120 bp
		PCR-R	GGGTATGGACAGATCTCAAAAAAAGCAC	
		*bap*-F	GAAAAGATGATGATAACATTAAAG	120 bp
		PCR-R	GGGTATGGACAGATCTCAAAAAAAGCAC	
	Sanger Sequencing	*icaA*-F	CCATATGGCTTACAACCTAACTAACG	1,328 bp
		*icaA*-R	GATATAGCGATAAGTGCTGTTTCTC	
		*icaD*-F	AGTCGCACTCTTTATTGATAGTCG	525 bp
		*icaD*-R	CCAGTGTGCTTACAGGCAATA	
		*bap*-F	CCAATGACACTGCTGTTGAATCTACG	1,090 bp
		*bap*-R	GCACGAATAAAGTTGACTTCCCAAG	
	Convantional PCR and RT-qPCR	*mec*A-F	CGTTACAGTGTCACTTTCAACAT	310 bp
		*mec*A-R	AACGATTGTGACACGATAGCC	
		*ica*A-F	GAGGTAAAGCCAACGCACTC	131 bp
		*ica*A-R	CCTGTAACTGCACCAAGTTT	
		*ica*D-F	ACCCAACGCTAAAATCATCG	211 bp
		*ica*D-R	GCGAAAATGCCCATAGTTTC	
		*bap*-F	GCAACACCACAAAGAACTATG	227 bp
		*bap*-R	CTGTTTCCAGAGTTTGCTCC	
		*rRNA*-F	ACGTGGATAACCTACCTATAAGACTGGGAT	150 bp
		*rRNA-R*	TACCTTACCAACTAGCTAATGCAGCG	
OLIGOS	*ica*A spacer for gene deletion	*ica*A-F	GAAAGTCGATTTACAAGAAAACAG	20 bp (315–335 bp of the coding Sequence)
		*ica*A-R	AAACCTGTTTTCTTGTAAATCGAC	
	*ica*D spacer for gene deletion	*icaD-*F	GAAATTAATAATCCAGTATACTGT	20 bp (upstream positions 63–83 bp of the coding Sequence)
		*icaD-*R	AAACACAGTATACTGGATTATTAA	
	*bap* spacer for gene deletion	*bap*-F	GAAAAGATGATGATAACATTAAAG	20 bp (374–394 of the coding Sequence)
		*bap-R*	AAACCTTTAATGTTATCATCATCT	

### 2.2 Molecular detection of *mec*A and biofilm-associated genes

Genomic DNA of the clinical MRSA strain was isolated using the Nucleospin Microbial DNA kit (Macherey-Nagel GmbH&Co KG, Düren, Germany). Bacterial cultures were grown in Luria Bertani (LB) broth (Merck KGaA, Darmstadt, Hessen, Germany) for 18 h. Cells were harvested by centrifugation, resuspended in elution buffer, and lysed using lysis buffer and proteinase K. Following clarification by centrifugation, genomic DNA was purified using a spin-column–based protocol, washed to remove impurities, eluted, and stored at −20 °C until use.

*mec*A-resistance gene and biofilm-associated genes (*icaA*, *icaD*, and *bap*) were identified in MRSA by conventional PCR. PCR cycling conditions for four genes (*icaA*, *icaD*, *bap*, and *mec*A) were as follows: 94 °C for 4 min (pre-denaturation), followed by 30 cycles of 94 °C for 45 s (denaturation), 63 °C for 45 s (annealing), and 72 °C for 1 min (extension), with a final extension at 72 °C for 1 min. The primer sequences listed in Table 1 were used.

### 2.3 Restriction digestion

The pCasSA plasmid was linearized by incubation with BsaI-HFv (NEB, Ipswich, MA, United States) at 37 °C for 15 min, followed by enzyme inactivation at 80 °C for 20 min. The linearized plasmid was separated by 1% agarose gel electrophoresis, purified using the Plus Gel Eluted Kit (GMbiolab, Taichung, Taiwan), and stored at −20 °C until use (Szeberényi, 2013).

### 2.4 Oligonucleotide ligation into plasmid

Oligos were designed using Benchling (Benchling, San Francisco, California, United States) to match the sticky ends of the linearized plasmid (Table 1). After phosphorylation with T4 polynucleotide kinase (NEB, Ipswich, MA, United States) and annealing, oligos were ligated into the plasmid using T4 DNA ligase (NEB, Ipswich, MA, United States). The ligation products were transformed into chemically competent *Escherichia coli* DH10B cells by heat shock (Chang et al., 2017) and selected on LB agar (Merck KGaA, Darmstadt, Hessen, Germany) containing kanamycin (50 μg/mL) (Provet, Istanbul, Türkiye). Plasmids were purified using the Monarch Plasmid Miniprep Kit (NEB, Ipswich, MA, United States) and stored at −20 °C until use.

### 2.5 Colony PCR

Colony PCR was performed to verify the targeted ligation by amplifying a 120 bp region using the primers listed in Table 1. PCR cycling conditions were as follows: 5 min at 95 °C for initial denaturation, followed by 30 cycles of denaturation at 95 °C for 30 s, annealing at 58 °C for 30 s, elongation at 72 °C for 45 s, and final elongation at 72 °C for 5 min (Mai et al., 2022).

### 2.6 Electroporation

A single colony of MRSA was cultured in Tryptic Soy Broth (TSB) (Oxoid, Basingstoke, Hampshire, United Kingdom) for 18 h and diluted into fresh TSB (1:100). Cultures were incubated at 30 °C with shaking until OD₆₀₀ reached 0.4, at which point they were rapidly cooled on dry ice, harvested by centrifugation, and washed twice with 0.5 M sucrose. The final pellet was resuspended in 1 mL of 0.5 M sucrose, aliquoted. Aliquots (50 μL) werestored at −80 °C until use (Chen et al., 2017).

Fifty microliters of electrocompetent cells were mixed with 2 μg of pCasSA plasmid and transferred into a 1-mm electroporation cuvette. Electroporation was performed using an electroporator (Eporator, Eppendorf, Hamburg, Germany) at 21 kV/cm for 5.0 ms. Then, the CRISPR-edited cells were recovered in TSB at 30 °C for 1.5 h and plated on TSA containing chloramphenicol (5 μg/mL) for selection (Chen et al., 2017).

### 2.7 Determination of expression level of *ica*A, *ica*D, and *bap* genes by quantitative real-time PCR (RT-qPCR)

Wild-Type (WT) and CRISPR-edited strains were cultured on TSA, and bacterial suspensions prepared in TSB were adjusted to 0.5 McFarland. Cultures were incubated at 37 °C with shaking until reaching ~4 McFarland, after which total RNA was isolated using the Total RNA Purification Kit (GMbiolab, Taichung, Taiwan), following the manufacturer’s instructions. RNA purification included enzymatic lysis, DNase I treatment, and spin column-based extraction. RNA was eluted in nuclease-free water, quantified spectrophotometrically (Beckman Coulter DU-780, Beckman Coulter Inc., Fullerton, CA, United States), normalized to 100 ng/μL, and stored at −80 °C.

cDNA was synthesized from total RNA using the OneScript Plus Reverse Transcriptase cDNA Synthesis Kit (Applied Biological Materials Inc., BC, Canada) with random primers. RT-qPCR was performed using 2 × Magic SYBR Mix (Procomcure Biotech Gmbh, Salzburg, Australia) in 96-well plates with gene-specific primers targeting *icaA*, *icaD*, and *bap*. The 16S rRNA gene was used as the internal control to normalize gene expression. Amplification was carried out using a LightCycler 480 system (Roche, Rotkreuz, Switzerland) under optimized thermal conditions. PCR cycling conditions were as follows: 5 min at 95 °C for initial denaturation, followed by 40 cycles of denaturation at 95 °C for 10 s, annealing at 55 °C for 15 s, and elongation at 72 °C for 20 s, followed by melting curve analysis. All reactions were carried out in triplicate. Threshold cycle (CT) values were calculated using the LC480 2 software program (Roche, Rotkreuz, Switzerland). CT values were used to determine the fold change in gene expression of CRISPR-edited strains relative to WT, using the delta–delta CT (ΔΔCT) method;ΔCT (sample) = CT (target gene)-CT (16S rRNA), ΔΔCT = ΔCT (edited strain)-ΔCT (WT), Fold Change in Expression = 2^-(ΔΔCT)^ (Rao et al., 2013).

### 2.8 Broth microdilution method

To assess the impact of biofilm-associated gene targeting, antimicrobial susceptibility to oxacillin (Sigma-Aldrich Chemie GmbH, Taufkirchen, Germany), ciprofloxacin (Atabay, Istanbul, Türkiye), and gentamicin (Himedia, Mumbai, Maharashtra, India) was evaluated by the broth microdilution method according to European Committee on Antimicrobial Susceptibility Testing (EUCAST) guidelines. Bacterial suspensions were adjusted to a 0.5 McFarland standard with sterile saline and diluted 1:100 in Mueller–Hinton broth (MHB) (Oxoid, Basingstoke, Hampshire, United Kingdom). Stock solutions of each antibiotic were prepared at 2,048 μg/mL in sterile distilled water and sterilized by membrane filtration. Two-fold serial dilutions of each antibiotic were prepared in U-bottom 96-well microtiter plates, followed by inoculation with bacterial suspensions. *S. aureus* ATCC 29213 was included as a quality control strain, and sterility and growth controls were incorporated in each assay. Plates were incubated at 37 °C for 18 h, and MIC values (mg/L) were defined as the lowest antibiotic concentration preventing visible growth. All experiments were performed in triplicate (International Organization for Standardization (ISO), 2019; European Committee on Antimicrobial Susceptibility Testing, 2025).

### 2.9 Disk diffusion method

Bacterial suspensions of WT and CRISPR-edited strains were adjusted to 0.5 McFarland turbidity and inoculated onto Mueller Hinton Agar (MHA) (Oxoid, Basingstoke, Hampshire, United Kingdom) using sterile swabs. Cefoxitin (Bioanalyse, Ankara, Türkiye), gentamicin (Bioanalyse, Ankara, Türkiye), and norfloxacin (Bioanalyse, Ankara, Türkiye) disks were placed on the agar surface. Plates were incubated at 37 °C for 18 h, after which inhibition zone diameters were measured (European Committee on Antimicrobial Susceptibility Testing, 2025).

### 2.10 Determination of biofilm capacities

The biofilm-forming capacity of WT and edited mutants were evaluated using the crystal violet microtiter plate assay as described by Stepanović et al. (2007), with minor modifications. Overnight cultures grown on TSA were adjusted to 0.5 McFarland and inoculated into 96-well flat-bottom plates containing TSB supplemented with 1% glucose (TSB-G). Following 24 h incubation at 37 °C, wells were washed with phosphate-buffered saline (PBS) (Oxoid, Basingstoke, Hampshire, United Kingdom) to remove planktonic cells, fixed with methanol (Sigma-Aldrich Chemie GmbH, Taufkirchen, Germany), and stained with 0.1% crystal violet (SigmaAldrich Chemie GmbH, Taufkirchen, Germany). The bound dye was solubilized with 95% ethanol, and absorbance was measured at 570 nm using a microplate reader (Varioskan Flash, Thermo Fisher Scientific Inc., Waltham, MA, United States). TSB-G served as the negative control, while *Enterococcus faecalis* ATCC 29212 was used as the positive control. All assays were performed in triplicate. The cut-off OD (ODc) was defined as three standard deviations above the mean OD of the negative control. Based on OD values, biofilm-forming capacity was categorized as follows: OD ≤ ODc, no biofilm production; ODc < OD ≤ 2 × ODc, weak biofilm producer; 2 × ODc < OD ≤ 4 × ODc, moderate biofilm producer; and OD > 4 × ODc, strong biofilm producer (Stepanović et al., 2007).

### 2.11 Sanger sequence analysis

Sanger sequencing was performed to analyze the *ica*A, *ica*D, and *bap* regions in WT and to confirm CRISPR-Cas9–mediated mutations in edited mutants. Genomic DNA from WT and CRISPR-edited strains was extracted using the Nucleospin Microbial DNA Kit (Macherey-Nagel, Düren, Germany), and CRISPR-targeted regions (~500–1,300 bp) were amplified by PCR using Q5 High-Fidelity DNA-Polymerase (NEB, Ipswich, MA, United States). PCR products were analyzed by Sanger sequencing, and obtained sequences were aligned with the NCBI reference sequence (Accession No. AY220730.1) (Aoki et al., 2024). The raw Sanger sequencing data were first visualized and quality-checked using SnapGene 8.2 (San Diego, CA, United States) to ensure the accuracy of the bases. The confirmed nucleotide sequences of the edited mutants were then aligned with the wild-type (WT) reference sequences obtained from the NCBI GenBank database (accession numbers: AY220730.1 and BA000017.4). The sequences were translated into their corresponding amino acid sequences using the SnapGene. By performing a protein-to-protein BLAST (BLASTp) alignment between the WT and mutant protein sequences, we were able to precisely identify the non-synonymous mutations and the resulting amino acid residues described in the manuscript.

### 2.12 Growth curve analysis

Growth kinetics of WT and edited mutants were assessed spectrophotometrically. Overnight bacterial cultures (WT and edited mutants) were adjusted to 0.5 McFarland standart. Bacterial suspensions were inoculated into LB broth (1:1,000). The broth cultures were incubated for 10 h with shaking at 37 °C and 200 rpm, and their optical density at 600 nm was measured spectrophotometrically at 2-h intervals for up to 10 h using a microplate reader (Varioskan Flash, Thermo Fisher Scientific Inc., Waltham, MA, United States). Growth rate differences between WT and CRISPR-edited strains were subsequently evaluated (Tagliaferri et al., 2020).

### 2.13 Statistical analysis

All experiments were performed in triplicate. Standard deviations were calculated using GraphPad-Prism-8 software (San Diego, CA, United States). Statistical-calculations were analyzed by one-way ANOVA, unpaired Student’s *t*-test. *p* < 0.05 was considered statistically significant.

## 3 Results

### 3.1 Antibiotic susceptibility profiles of clinical MRSA isolates

The identification of MRSA isolate were detected by MALDI-TOF MS using the VITEK MS Prime (Biomerieux, Lyon, France). Additionally, antimicrobial susceptibility testing was performed with the VITEK-2 compact automated system (Biomerieux, Lyon, France). The antibiotic susceptibility profile and the specimen type obtained clinical MRSA isolate were presented in Table 2. A clinical MRSA strain was isolated from a blood sample of a patient hospitalized in the Cardiovascular Surgery Service. As detailed in Table 1, the isolate was exhibited resistance to erythromycin, gentamicin, clindamycin, ciprofloxacin, penicillin, methicillin, and tetracycline, while remaining susceptible to trimethoprim/sulfamethoxazole, linezolid, teicoplanin, and tigecycline.

**Table 2:** The antibiotic susceptibility profile of the clinical isolate.

Strain	Source (patient sample)/where isolated	E	GN	DA	CIP	SXT	LZ	P	FOX	TEC	TET	TGC
MRSA	Peripheral blood culture/cardiovascular surgery service	R	R	R	R	S	S	R	R	S	R	S

S, Susceptible; R, Resistant; E, Erythromycin; GN, Gentamicin; DA, Clindamycin; CIP, Ciprofloxacin; SXT, Trimethoprim/Sulfamethoxazole; LZ, Linezolid; P, Penicillin; FOX, Methicillin; TEC, Teicoplanin; TET, Tetracycline; TGC, Tigecycline.

### 3.2 Detection of resistant gene and biofilm-associated genes and verification of CRISPR-

#### 3.2.1 Cas9 plasmid construction

**Figure 1:**
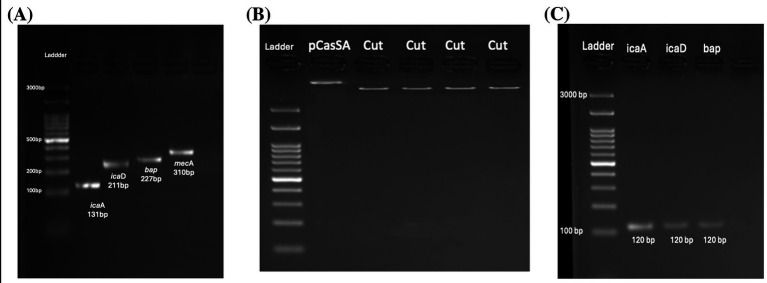
**(A)** *mecA*, *icaA*, *icaD*, and *bap* genes in clinical MRSA isolate with conventional PCR. **(B)** Restrictional digestion agarose gel electrophoresis image (Cut: pCasSA linearised with BsaI). **(C)** Colony PCR agarose gel electrophoresis image.

The *mec*A, *ica*A, *ica*D, and *bap* genes in MRSA isolate were detected by conventional-PCR. The gel image was shown in Figure 1A. The detection of all four genes confirmed the methicillin-resistant and biofilm-forming phenotype of the isolate, providing the molecular basis for the subsequent CRISPR-Cas9-mediated targeting strategy.

The pCasSA was digested at positions 5,774. and 5,795. using BsaI. This generated a linear plasmid with sticky ends, facilitating the ligation of target-specific oligonucleotides. The gel electrophoresis image of the pCasSA and the linearized (cut-pCasSA) plasmid is shown in Figure 1B. Agarose gel electrophoresis revealed a single, clear band corresponding to the linearised plasmid at the predicted length. This was clearly distinguishable from the supercoiled and relaxed circular forms of the undigested pCasSA (Figure 1B). The absence of additional bands confirmed complete and specific digestion by BsaI, with no evidence of partial digestion or non-specific cleavage. These results verified that the plasmid was successfully prepared for subsequent oligonucleotide ligation steps.

To confirm the ligation, the 120 bp regions within pCasSA-*ica*A*spacer*, pCasSA*-icaD*spacer, and pCasSA-*bap*_spacer—which contain the oligonucleotides targeting the *ica*A, *ica*D, and *bap* regions—were amplified using Colony PCR. The presence of these amplified fragments was evaluated through agarose gel electrophoresis, and the results are presented in Figure 1C.

### 3.3 qPCR

**Figure 2:**
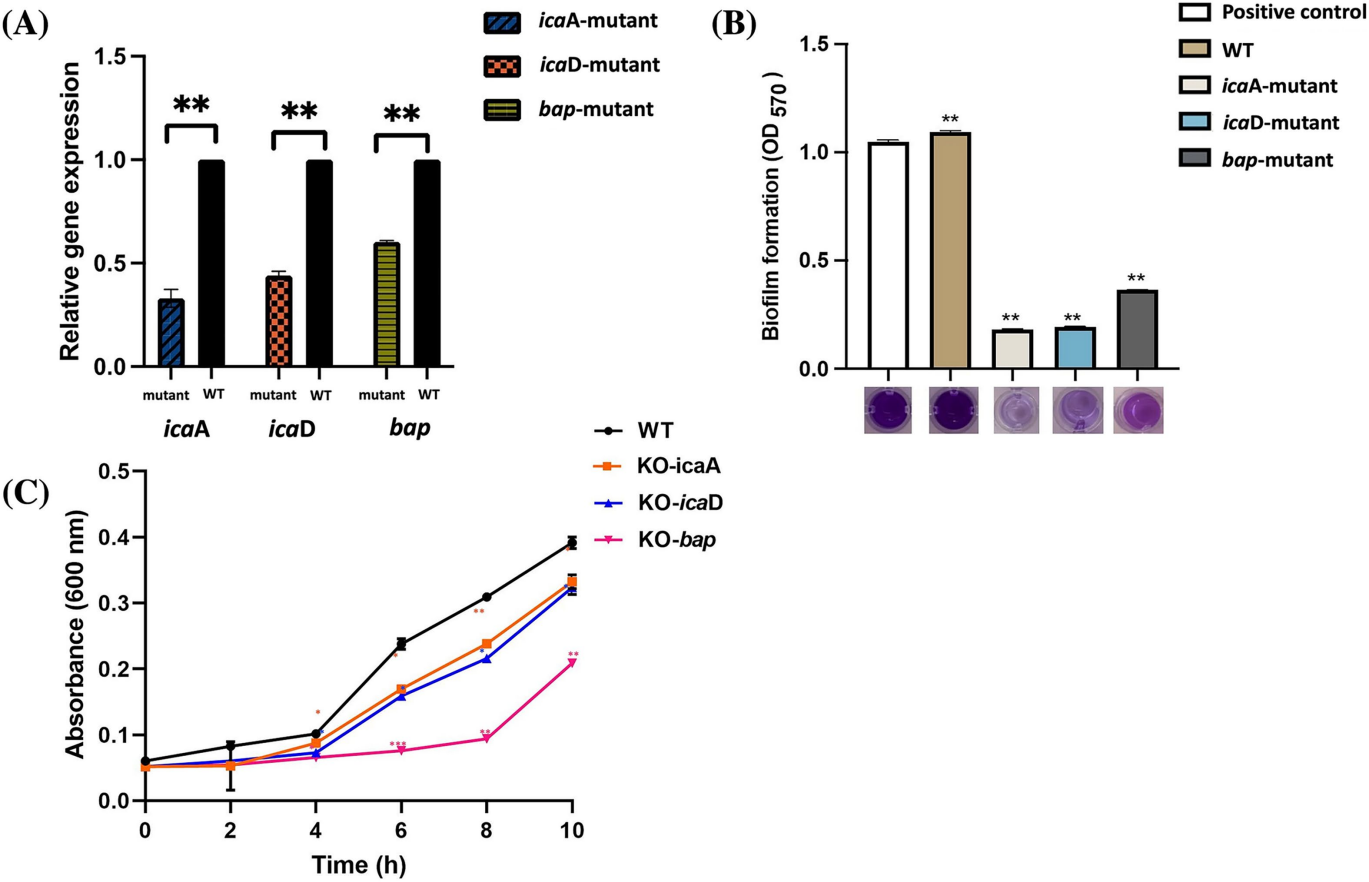
**(A)** Changes in the expression ratio of related genes in RT-qPCR (WT: Wild type, **: *p* < 0.01). **(B)** Biofilm formation of control, WT, and mutant-stains (WT, Wild type, **: *p* < 0.01). **(C)** Growth curves of WT and *icaA*-mutant, *icaD*-mutant, and *bap*-mutant strains (*: *p* < 0.05, **: *p* < 0.01, ***: *p* < 0.001).

The expression levels of *ica*A, *ica*D, and *bap* genes in CRISPR-Cas9-edited mutant strains were quantitatively assessed by RT-qPCR and compared to the WT strain. The changes in gene expression between edited mutants and WT strain are illustrated in Figure 2A. RT-qPCR analysis revealed significant downregulation of all three biofilm-associated genes in the edited mutant strains compared to the WT (*p* < 0.01). The *ica*A-mutant exhibited the most pronounced reduction in gene expression, with a 3.3-fold decrease in icaA transcript levels relative to the WT strain. This substantial downregulation is consistent with the effective CRISPR-Cas9-mediated disruption of the *ica*A locus, thereby impairing PIA synthesis, which is essential for intercellular adhesion and mature biofilm formation. Additionally, the edited mutant displayed a 2.3.-fold reduction in *ica*D gene expression, a 1.7-fold reduction in *bap* gene expression, compared to the WT strain.

### 3.4 Broth microdilution and disk diffusion method

The effect of CRISPR-Cas9-mediated targeting of *ica*A, *ica*D, and *bap* genes on the antimicrobial susceptibility profile of MRSA is summarized in Tables 3, 4. Interpretive criteria were based on EUCAST guidelines. For broth microdilution: oxacillin MIC >2 mg/L indicated methicillin resistance; ciprofloxacin MIC ≤0.001 mg/L susceptible, >2 mg/L resistant; gentamicin MIC ≤2 mg/L susceptible, >2 mg/L resistant. For disk diffusion: cefoxitin zone diameter ≥22 mm susceptible, <22 mm resistant; norfloxacin ≥17 mm susceptible, <17 mm resistant; gentamicin ≥18 mm susceptible, <18 mm resistant.

**Table 3:** Results of broth microdilution method.

Strain	Minimum inhibitory concentration (MIC) (mg/L)					
	Oxacillin	Categories	Ciprofloxacin	Categories	Gentamicin	Categories
MRSA(WT)	256	R	>512	R	>512	R
*ica*A-mutant	4	R	4	R	4	R
*ica*D-mutant	16	R	8	R	4	R
*bap*-mutant	64	R	8	R	8	R

S, Susceptible; R, Resistant; WT, Wild Type. Interpretive criteria were based on EUCAST guidelines. For broth microdilution: oxacillin MIC >2 mg/L indicated methicillin resistance; ciprofloxacin MIC ≤0.001 mg/L susceptible, >2 mg/L resistant; gentamicin MIC ≤2 mg/L susceptible, >2 mg/L resistant.

**Table 4:** Results of disk diffusion method.

Strain	Diameter of inhibition zone (mm)					
	Cefoxitin	Categories	Norfloxacin	Categories	Gentamicin	Categories
MRSA(WT)	10	R	6	R	6	R
*ica*A-mutant	20	R	14	R	16	R
*ica*D-mutant	18	R	12	R	11	R
*bap*-mutant	15	R	10	R	10	R

S, Susceptible; R, Resistant; WT, Wild Type. Interpretive criteria were based on EUCAST guidelines. For disk diffusion: cefoxitin zone diameter ≥22 mm susceptible, <22 mm resistant; norfloxacin ≥17 mm susceptible, <17 mm resistant; gentamicin ≥18 mm susceptible, <18 mm resistant.

The wild-type isolate exhibited resistance to all tested antibiotics, with MIC values of 256 mg/L for oxacillin and >512 mg/L for both ciprofloxacin and gentamicin. Following CRISPR-Cas9-mediated gene editing, MIC values were substantially reduced across all mutant strains (Table 3). The most dramatic shift was observed in the *icaA*-mutant, where the oxacillin MIC decreased 64-fold (from 256 to 4 mg/L). Furthermore, the ciprofloxacin and gentamicin MICs for the *icaA*-mutant dropped at least 128-fold, both reaching 4 mg/L. Similar trends were noted for the *icaD* and *bap* mutants; the ciprofloxacin MIC decreased 64-fold (to 8 mg/L) in both strains. In the *icaD*-mutant, MIC values were 16 mg/L for oxacillin (16-fold reduction), 8 mg/L for ciprofloxacin (>64-fold reduction), and 4 mg/L for gentamicin (>128-fold reduction). In the *bap*-mutant, MIC values were 64 mg/L for oxacillin (4-fold reduction), 8 mg/L for ciprofloxacin (>64-fold reduction), and 8 mg/L for gentamicin (>64-fold reduction) (Table 3). Complementary results from the disk diffusion method (Table 4) supported these findings. Based on the disk diffusion test results, WT isolate exhibited inhibition zone diameters of 10 mm for cefoxitin, 6 mm for norfloxacin, and 6 mm for gentamicin. The inhibition zone diameters indicate that the WT isolate exhibits resistance to all three antibiotics. In the gene-edited isolates, the zone diameters were 20 mm, 14 mm, 16 mm in *ica*A-mutant; 18 mm, 12 mm, 11 mm in *ica*D-mutant; 15 mm, 10 mm and 10 mm in *bap*-mutant, respectively (Table 4). All edited isolates remained in the resistant category for all agents tested.

CRISPR-Cas9-mediated suppression of biofilm-associated genes was further supported by marked phenotypic changes: oxacillin, ciprofloxacin, and gentamicin MIC values were significantly reduced, and cefoxitin, norfloxacin, and gentamicin zone diameters were significantly increased (Tables 3, 4). Among the targeted genes, *ica*A knockout produced the most pronounced phenotypic effect, suggesting a central role of PIA synthesis in biofilm-mediated antibiotic resistance. While edited mutants remained clinically resistant, the significant reductions in MIC values and increased inhibition zone diameters indicate partial attenuation of resistance phenotypes.

### 3.5 Determination of biofilm capacities

The effect of CRISPR-mediated silencing of *ica*A, *ica*D, and *bap* genes on MRSA biofilm was studied using the quantitative microplate method. While the WT strain exhibited strong biofilm-forming capacity, the *ica*A-mutant and *ica*D-mutant variants were identified as weak biofilm producers (Figure 2B). In comparison to the WT, suppression of the *bap* gene resulted in a moderate level of biofilm production. The wild-type MRSA displayed a strong biofilm-positive phenotype, with a mean optical density (OD_595_) of 1.096 ± 0.0003. Following targeted gene editing, the most profound reduction was observed in the *icaA*-mutant, where biofilm formation decreased by approximately 91.3% (OD_595_: 0.183 ± 0.002) This was followed by the *icaD*-mutant and *bap*-mutant, which showed reductions of 90.4% (OD_595_: 0.192 ± 0.0004) and 73.1% (OD_595_: 0.366 ± 0.0006), respectively (Figure 2B).

### 3.6 Sanger sequences analysis

**Figure 3:**
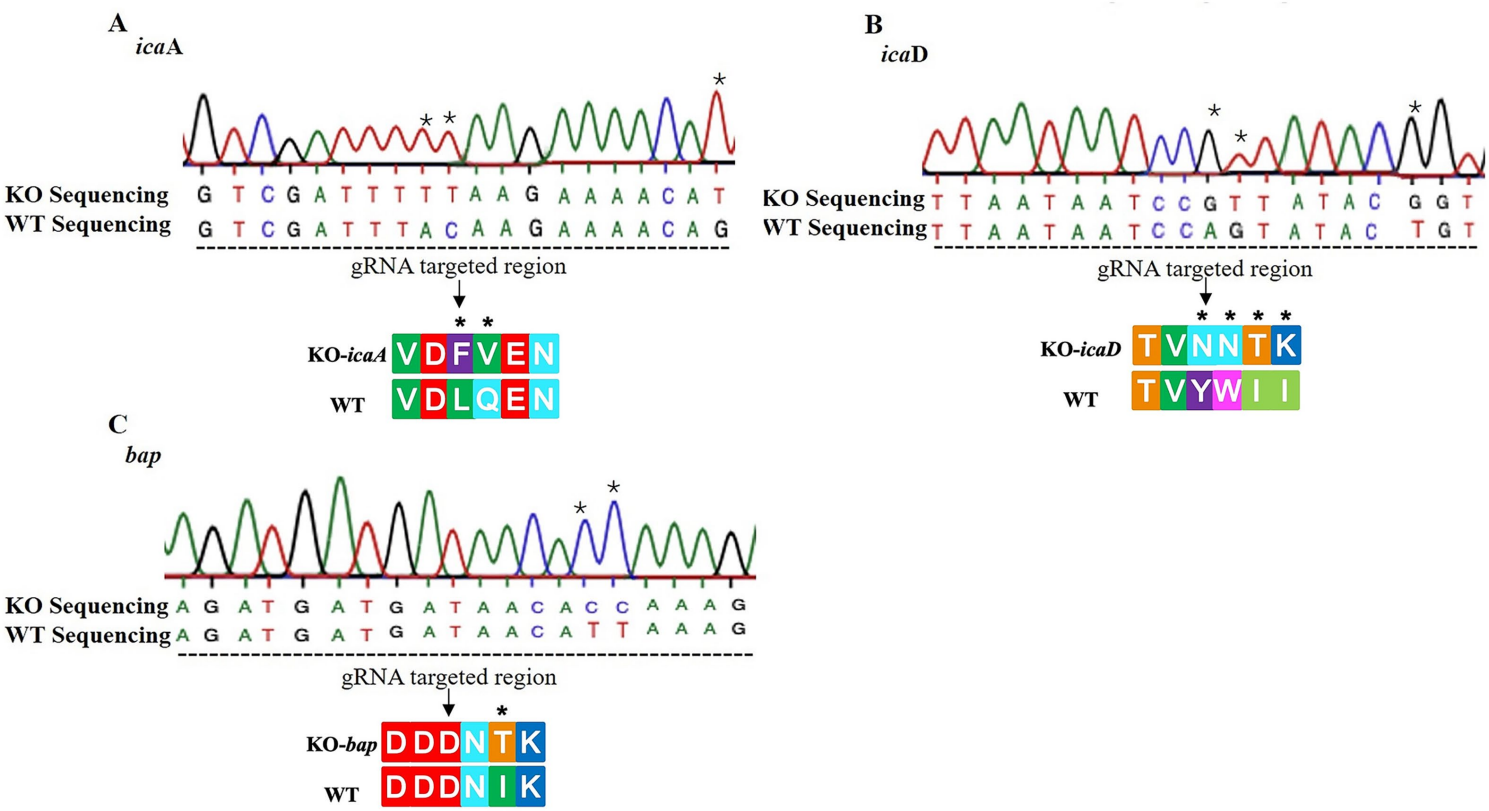
Mutations detected in mutant strains. **(A)** In the *icaA*-mutant, positions 324, 325, and 355 of the *icaA* gene occurs A→T, C→T, G→T mutations. **(B)** In the *icaD*-mutant, positions −74 and −75 of the *icaD* gene occurred G→T, A→G mutations. **(C)** In the *bap*-mutant, positions 389 and 390 of the *bap* gene occurred T→C point mutations.

Sanger sequencing was performed to confirm CRISPR-Cas9-mediated mutations in the icaA, icaD, and bap target regions of the edited strains relative to the WT. The mutations identified in the edited strains, in comparison to the WT strain, are shown in Figure 3. Sanger sequencing revealed point mutations as well as small insertions or deletions (indels) within the target regions of all edited strains. No complete gene deletions were detected in any of the edited strains. The observed genetic alterations are consistent with error-prone non-homologous end joining (NHEJ), the predominant DNA repair pathway activated following Cas9-induced double-strand breaks. Amino acid sequence analysis of the *ica*A-mutant, *ica*D-mutant, and *bap*-mutant strains revealed multiple point mutations resulting from gene editing (Figure 3; Table 5).

**Table 5:** Nucleotide and amino acid changes in CRISPR-Cas9-edited KO strains relative to the wild-type MRSA isolate.

Target gene	Nucleotide position	Nucleotide change	Amino acid change
*ica*A	324, 325, 355	AT, CT, GT mutations	Gln to Val (Q→V), Leu to Phe (L→F)
*ica*D	−74, −75, −81	GT, AG, TG mutations	Tyr to Asn (Y→N), Trp to Asn (W→N), Ile to Thr (I→T), Ile to Lys (I→K)
*bap*	389, 390	TC point mutation	Ile to Thr (I→T)

In the *ica*A-mutant, nucleotide changes at positions 324. (A→T), 325. (C→T), and 355. (G→T) resulted in two amino acid substitutions: glutamine to valine (Q → V) and leucine to phenylalanine (L → F). These substitutions are predicted to cause protein misfolding, thereby impairing IcaA function and consequently reducing PIA synthesis and biofilm formation (Figure 3A, Table 5). In the icaD-mutant, nucleotide changes at positions −74 (G→T), −75 (A→G), and −81 (T→G) resulted in four amino acid substitutions: tyrosine to asparagine (Y → N), tryptophan to asparagine (W → N), isoleucine to threonine (I → T), and isoleucine to lysine (I → K). The accumulation of multiple amino acid changes in IcaD is likely to substantially disrupt protein conformation and function, which is consistent with the observed 2.3-fold reduction in *ica*D gene expression and 5.6-fold reduction in biofilm formation (Figure 3B; Table 5). In the *bap*-mutant, a point mutation at nucleotide positions 389 (T→C) and 390 (T→C) resulted in a single amino acid substitution: isoleucine to threonine (I → T). Although this represents a single residue change, it was sufficient to alter Bap protein function, as evidenced by the 1.7-fold reduction in bap gene expression and the 3-fold decrease in biofilm formation compared to the WT strain (Figure 3C; Table 5). These changes are likely to affect the structure and function of proteins and may be associated with the reduction in biofilm formation of edited mutants. Mutations introduced via CRISPR-Cas9 effectively silenced biofilm genes, leading to reduced gene expression and increased antibiotic susceptibility. As such, CRISPR-Cas9 was effectively utilized to target biofilm-related genes and induce the desired genetic and functional outcomes.

### 3.7 Growth curves

Growth kinetics of the WT and CRISPR-edited mutant strains (icaA-mutant, icaD-mutant, and bap-mutant) were assessed spectrophotometrically at 600 nm over a 10-h incubation period. No significant differences in growth were observed among the strains during the early growth phase (0., 2., and 4.h), indicating that CRISPR-Cas9-mediated gene editing did not affect initial bacterial proliferation. A discernible shift in growth kinetics became apparent in the spectrophotometric readings from the 6th hour onward (Figure 2C). At the 6th hour, the bap-mutant exhibited the most pronounced reduction in growth rate, with a 3.14-fold decrease compared to the WT strain. In contrast, the *icaA*-mutant and *ica*D-mutant demonstrated more moderate reductions of 1.4-fold and 1.5-fold, respectively, at the same time point. At the 8th hour, the *bap*-mutant exhibited a 3.3-fold reduction in growth rate, relative to the WT. The *ica*A-mutant and *ica*D-mutant maintained similar reductions of 1.3-fold and 1.43-fold, respectively, consistent with the patterns observed at the 6th hour. By the 10th hour, growth rate differences between the mutant and WT strains were attenuated but remained detectable. The bap-mutant exhibited a 1.88-fold reduction, while both the *ica*A-mutant and *ica*D-mutant demonstrated 1.18-fold ans 1.21-fold reductions compared to the WT strain (Figure 2C). During the period under observation, the cell density of the *bap*-mutant was consistently lower than that of both the WT and the other edited strains, suggesting that bap plays a broader role in bacterial physiology beyond biofilm formation, potentially influencing fundamental cellular processes such as proliferation and metabolic activity.

## 4 Discussions

In the present study, we applied CRISPR-Cas9 technology as an efficient gene-editing strategy to inhibit biofilm formation in MRSA. The present study demonstrated that CRISPR-Cas9-mediated disruption of *ica*A, *ica*D, and *bap* in a clinical MRSA isolate resulted in marked reductions in biofilm-associated gene expression, biofilm formation, and antimicrobial resistance phenotypes. A review of the current literature reveals a gap in studies that specifically use genome-editing tools to target biofilm-associated genes in MRSA. CRISPR-Cas offers both high sensitivity and precision in gene targeting, allowing for the direct observation of functional changes resulting from specific gene a single plasmid system (pCasSA) encoding both Cas9 and sgRNA (Ates et al., 2024), we successfully edited *S.aureus* genomic loci, consistent with prior findings demonstrating the effectiveness of this system in introducing mutations (Chen et al., 2017; Gu et al., 2018). Mutations in the *ica*A, *ica*D, and *bap* genes were confirmed by Sanger sequencing (Figure 3). The *ica* operon, particularly *ica*A and *ica*D, plays a crucial role in biofilm development through the synthesis of PIA, a core component of the EPS matrix (Peng et al., 2022). These genes are typically found in biofilm-forming *S.aureus* isolates of varying capacities and are generally absent in non-biofilm producers (Armoon et al., 2024). In our study, the MRSA isolate demonstrated strong biofilm-forming capacity by crystal violet staining, and the presence of *ica*A, *ica*D, and *bap* genes was confirmed by PCR.

Biofilm-associated antibiotic resistance is multifactorial, involving limited drug penetration, alterations in the local microenvironment, and phenotypic/metabolic adaptations of the bacteria (Sharif and Yadav, 2025; Grooters et al., 2024). To our knowledge, no previous study targeting biofilm-associated genes via CRISPR-Cas9 in MRSA has systematically evaluated the resulting changes in antimicrobial susceptibility using both broth microdilution and disk diffusion methods. This dual assessment represents a methodological strength of the present study and provides a more comprehensive phenotypic characterization than prior work. In this study, we used both disk diffusion and broth microdilution methods to evaluate resistance to methicillin, ciprofloxacin, and gentamicin following CRISPR-Cas9 silencing of *ica*A, *ica*D, and *bap*. Notably, the MIC for oxacillin decreased from 256 mg/L in the WT isolate to 4 mg/L in the KO isolate. In parallel, the cefoxitin inhibition zone increased from 10 mm in the WT to 22 mm in the edited mutants. Similar trends were observed for ciprofloxacin and gentamicin, with increased zone diameters and reduced MIC values. In our study, the missense mutations resulting from amino acid changes significantly impaired gene function. The amino acid changes in the edited mutants led to functional inactivation of the targeted genes. In particular, the glutamine-to-valine and leucine-to-phenylalanine substitutions in icaA are predicted to affect protein folding, which may contribute to the observed reduction in biofilm formation. Similarly, multiple amino acid changes in *ica*D and a single change in bap may disrupt protein conformation, thereby affecting biofilm formation and stability. CRISPR-induced missense mutations can disrupt gene function by causing protein misfolding (Chavez et al., 2023). These changes are likely to affect the structure and function of proteins and may be associated with the reduction in biofilm formation of edited mutants. Mutations introduced via CRISPR-Cas9 effectively silenced biofilm genes, leading to reduced gene expression and increased antibiotic susceptibility. As such, CRISPR-Cas9 was effectively utilized to target biofilm-related genes and induce the desired genetic and functional outcomes. These findings demonstrate that biofilm suppression significantly enhances antibiotic susceptibility. While biofilm inhibition alone does not fully reverse resistance, it contributes meaningfully to reducing resistance levels and potentially improving treatment efficacy.

The present findings are consistent with and supported by a growing body of literature employing CRISPR-based technologies to investigate and disrupt biofilm-associated gene functions in *S. aureus* and MRSA. Jain et al. (2022) utilized a CRISPR-Cas9 base-editing approach to generate *maz*E and *maz*F mutants in a clinical ST239 MRSA isolate. The *maz*EF toxin-antitoxin system, which regulates *cid*A-mediated cell death and extracellular DNA release, plays a key role in biofilm formation. Disruption of this system significantly altered biofilm development and attenuated antibiotic resistance phenotypes, including reduced susceptibility to oxacillin, daptomycin, and vancomycin (Jain et al., 2022). In line with these findings, our study demonstrates that CRISPR-Cas9-mediated disruption of i*ca*A, *ica*D, and *bap* resulted in gene-specific reductions in both biofilm formation and MIC values. The most pronounced effect was observed in the *ica*A mutant, which exhibited a sixfold decrease in biofilm formation and a 64-fold reduction in oxacillin MIC. These findings highlight the central role of PIA synthesis in biofilm-mediated resistance in this clinical isolate. The broader relevance of CRISPR-based biofilm gene targeting in *S. aureus* is further supported by the work of Kouvela et al. (2025). Using the same pCasSA genome editing platform as in the present study, the authors ablated a tRNA^Gly^ gene copy in *S. aureus* RN4220. This intervention revealed unexpected downstream effects on cell wall integrity, antibiotic susceptibility, and biofilm formation, highlighting that targeted genomic disruption in *S. aureus* can lead to pleiotropic phenotypic outcomes beyond the primary target (Kouvela et al., 2025). In a complementary line of research, Long et al. (2022) demonstrated, through multi-omics analyses and CRISPR-mediated genetic manipulation, that elasnin effectively eradicates pre-formed MRSA biofilms. This effect is achieved by repressing key virulence regulons, particularly *sar*A, *sar*Z, and *RNA*III, leading to matrix disruption and increased susceptibility of released cells to the *β*-lactam antibiotic penicillin G (Long et al., 2022). These findings are consistent with the partial attenuation of antibiotic resistance phenotypes observed in the edited mutants of our study. It is worth noting that both Kouvela et al. (2025) and Long et al. (2022) employed the pCasSA genome editing platform (Kouvela et al., 2025; Long et al., 2022), the same system used in our study. This methodological consistency enhances the comparability of findings across studies and supports the reproducibility and robustness of CRISPR-Cas9-mediated gene editing in *S. aureus*. Garmendia-Antoñana et al. (2026) demonstrated that phage-delivered CRISPR-Cas9 modules (ePICIs) exhibit potent bactericidal activity against *S. aureus* in a murine mastitis model. They also showed that the composition of the biofilm extracellular matrix-specifically the proteinaceous Bap matrix versus the PIA/PNAG exopolysaccharide matrix-critically influences CRISPR delivery efficiency (Garmendia-Antoñana et al., 2026). This finding is particularly relevant to our study, as disruption of the *bap* gene resulted in the most modest phenotypic changes, suggesting that Bap-mediated matrix components may provide partial protection against genetic editing tools. Collectively, these studies support the premise that CRISPR-Cas9-based targeting of biofilm structural and regulatory genes is a viable and precise anti-virulence strategy in MRSA. The phenotypic outcomes of such interventions depend on the specific gene targeted and the genetic background of the clinical isolate. In addition, the composition of the biofilm matrix plays a critical role in shaping these outcomes. Several studies have investigated the role of biofilm-associated genes in bacterial growth dynamics by targeting specific genes in different bacterial species. In a study conducted by Zhong et al. (2021), which investigated LuxR family regulators associated with biofilm formation and quorum sensing in *Vibrio parahaemolyticus*, it was demonstrated that the growth rates of Knock-out *lux*R strains were significantly reduced compared to the WT strain. This finding highlights the potential role of *Lux*R-type transcriptional regulators in modulating bacterial growth dynamics (Zhong et al., 2021). Several CRISPR-Cas9 studies in other bacterial species have successfully targeted biofilm-related genes, such as luxS in *E. coli* (Zuberi et al., 2017a), cyclic-di-GMP pathway regulators in *Pseudomonas fluorescens* (Noirot-Gros et al., 2019), *omp*A in *Cedecea neteri* (Hegde et al., 2019), and *fim*H in *E. coli* (Zuberi et al., 2017b). Notably, these studies predominantly employed CRISPRi-a transcriptional repression strategy that suppresses gene expression without introducing permanent genomic alterations-and were conducted in Gram-negative organisms that lack the *ica* operon. The present study differs in three key respects: it applies CRISPR-Cas9 mediated editing, which generates stable, heritable mutations confirmed by Sanger sequencing; it targets the *ica* operon and *bap* gene, which are central to biofilm formation in Gram-positive staphylococci; and it extends the phenotypic analysis to include quantitative assessment of antibiotic susceptibility changes following gene disruption. The deletion of biofilm-associated genes (*ica*A, *ica*D, and *bap*) in MRSA was associated with a reduction in bacterial growth rate, most notably in the *bap*-mutant. These results support that regulators and structural components involved in biofilm formation may also influence fundamental cellular processes such as proliferation and metabolic activity. In another study, mutations in the biofilm-associated *car*B gene of *Xanthomonas citri* were shown to lead to a significant reduction in bacterial growth rate (Zhuo et al., 2015). This observation is consistent with our findings. These findings suggest that biofilm-associated genes may also play roles in bacterial physiology, particularly in processes essential for optimal proliferation. To date, there is limited evidence regarding the potential effects of CRISPR-mediated disruption of biofilm-associated genes (*icaA*, *icaD*, and *bap*) on the growth kinetics and bacterial fitness. The functional interaction between biofilm gene suppression and bacterial fitness remains largely unexplored. Our findings may provide preliminary insights into this field and provide a basis for future investigations.

This study has notable strengths. The significant downregulation in *icaA*, *icaD*, and *bap* expression confirm the efficiency and specificity of the CRISPR-Cas9 system in targeted genes. The significant reduction in gene expression suggests that CRISPR-Cas9-mediated gene editing may serve as an anti-virulence strategy to attenuate biofilm formation in *S. aureus*, though further studies are needed to evaluate its translational potential in clinically relevant models. These findings underscore the potential of CRISPR-Cas9 gene editing as a targeted anti-virulence approach against biofilm-associated MRSA infections. Down-regulation of *ica*A, *ica*D, and *bap* genes in the MRSA isolate led to a notable decrease in biofilm formation in the edited strains. These findings highlight the high sensitivity and efficiency of the CRISPR-Cas9-mediated genome editing approach in modulating the expression of biofilm-associated genes. Our study further showed that suppression of PIA synthesis via gene editing led to decreased EPS formation, directly reducing the biofilm-forming capacity of the MRSA isolate. This functional disruption of the biofilm matrix significantly altered both phenotypic resistance and virulence-associated behavior. Among the three targeted genes, *ica*A disruption produced the most pronounced effects across all measured outcomes, suggesting a central role for PIA synthesis in biofilm-mediated resistance in this isolate. CRISPR-Cas9-mediated biofilm gene disruption meaningfully attenuates antimicrobial resistance phenotypes, it is insufficient on its own to achieve clinical susceptibility. These findings highlight how CRISPR-Cas9-mediated biofilm disruption can weaken bacterial defenses. The combination of biofilm suppression and resistance gene editing may represent a synergistic strategy for attenuating antimicrobial resistance phenotypes in MRSA. This study demonstrates that genomic interference targeting biofilm may enhance antibiotic efficacy and provide a complementary strategy in the treatment of biofilm-associated MRSA infections.

The use of a clinically derived MRSA isolate, rather than a laboratory-adapted strain, enhances the clinical relevance of the findings. The combination of molecular (qPCR, Sanger sequencing), phenotypic (crystal violet assay), and susceptibility testing (broth microdilution and disk diffusion) approaches provides a comprehensive characterization of the editing outcomes. From a translational perspective, These findings suggest that CRISPR-Cas9-based gene editing of biofilm-associated genes could serve as a complementary approach alongside conventional antibiotics. This strategy may help reduce the effective antibiotic dose required to treat biofilm-associated MRSA infections In addition, it could be further developed by integrating nanoparticle-based or bacteriophage-mediated delivery systems for targeted application. (Mayorga-Ramos et al., 2023; Zuberi et al., 2024; Saffari Natanzi et al., 2025).

The use of a single clinical MRSA isolate is a limitation of the study. Biofilm gene content, regulatory networks, and CRISPR editing efficiency may vary considerably among isolates (Patel and Rawat, 2023; Dyzenhaus et al., 2023; Mikkelsen et al., 2023; Chan et al., 2024). Accordingly, the results should be interpreted as proof-of-concept, and future studies employing a broader panel of clinical isolates are needed to assess the generalizability of this approach. Despite the promising findings of this study, several challenges must be addressed before CRISPR-Cas9-based anti-virulence strategies can be translated into clinical practice. A primary concern is the efficient and safe delivery of CRISPR components into target bacterial cells *in vivo* (Sioson et al., 2021). Phage-mediated delivery systems and lipid nanoparticles have been proposed as promising vehicles for in vivo CRISPR delivery; however, their efficacy, specificity, and safety profiles require further evaluation in preclinical and clinical models (Khambhati et al., 2022; Mayorga-Ramos et al., 2023). Additionally, the potential for off-target editing, the emergence of CRISPR-resistant bacterial variants, and the regulatory complexities surrounding gene-editing therapeutics represent significant hurdles that must be systematically investigated (Mayorga-Ramos et al., 2023). The present study therefore serves as a proof-of-concept demonstration of CRISPR-Cas9-mediated biofilm gene disruption in a clinical MRSA isolate, rather than a direct clinical intervention, and further studies in animal models and eventually clinical settings are warranted to establish its translational potential.

## 5 Conclusion

In conclusion, to our knowledge, this study represents one of the first applications of CRISPR-Cas9 technology for the targeted suppression of biofilm-associated genes in a clinical MRSA isolate. In this study, we combined an anti-virulence approach with CRISPR-Cas9 gene-editing technology to demonstrate that anti-biofilm activity can be effectively harnessed to target MRSA. Our findings support the potential of this approach in reducing biofilm formation, thereby offering a novel strategy for the treatment of biofilm-associated infections. From a translational perspective, the findings of this study may have important implications for the development of next-generation antimicrobial strategies. CRISPR-Cas9-based biofilm gene disruption could potentially be integrated into phage-delivered or nanoparticle-based therapeutic platforms, enabling targeted in vivo delivery to infected person. Furthermore, combining CRISPR-mediated anti-virulence approaches with conventional antibiotics may represent a synergistic strategy to lower effective therapeutic doses and reduce the risk of resistance development. We recommend the use of in vivo infection models in future studies to further validate the therapeutic efficacy of CRISPR-based biofilm inhibition in MRSA infections. Consistent with prior research targeting biofilm formation in various bacterial species, our study underscores the transformative potential of CRISPR-Cas technology in infectious disease therapeutics. As personalized medicine continues to advance, pathogen-specific CRISPR therapeutics tailored to the genetic profile of clinical isolates could offer a transformative alternative to broad-spectrum antibiotics, ultimately contributing to the global effort to combat antimicrobial resistance. Looking ahead, the development of personalized or pathogen-specific CRISPR therapeutics represents a promising direction for the treatment of resistant infections. These advancements may pave the way for significant breakthroughs in the fight against antimicrobial resistance. These findings contribute to the growing body of knowledge supporting CRISPR as a precise and powerful tool for targeting bacterial virulence. Ultimately, this work supports the future development of pathogen-specific, gene-editing-based antimicrobial therapies as a novel avenue in the fight against antibiotic resistance.

## Data Availability

The data presented in this study are publicly available. The data can be found at: https://www.ncbi.nlm.nih.gov/genbank, PZ351582 (for icaA), PZ351583 (for icaD) and PZ351584 (for bap).
